# Assessment of normal myelination in infants and young children using the T1w/T2w mapping technique

**DOI:** 10.3389/fnins.2023.1102691

**Published:** 2023-02-28

**Authors:** Elena Filimonova, Evgenia Amelina, Aleksandra Sazonova, Boris Zaitsev, Jamil Rzaev

**Affiliations:** ^1^Federal Center of Neurosurgery, Novosibirsk, Russia; ^2^Department of Neurosurgery, Novosibirsk State Medical University, Novosibirsk, Russia; ^3^Stream Data Analytics and Machine Learning Laboratory, Novosibirsk State University, Novosibirsk, Russia; ^4^Department of Neuroscience, Institute of Medicine and Psychology, Novosibirsk State University, Novosibirsk, Russia

**Keywords:** myelination, magnetic resonance imaging, T1w/T2w mapping, infants, children

## Abstract

**Background:**

White matter myelination is a crucial process of CNS maturation. The purpose of this study was to validate the T1w/T2w mapping technique for brain myelination assessment in infants and young children.

**Methods:**

Ninety-four patients (0–23 months of age) without structural abnormalities on brain MRI were evaluated by using the T1w/T2w mapping method. The T1w/T2w signal intensity ratio, which reflects white matter integrity and the degree of myelination, was calculated in various brain regions. We performed a Pearson correlation analysis, a LOESS regression analysis, and a 2^nd^ order polynomial regression analysis to describe the relationships between the regional metrics and the age of the patients (in months).

**Results:**

T1w/T2w ratio values rapidly increased in the first 6–9 months of life and then slowed thereafter. The T1w/T2w mapping technique emphasized the contrast between myelinated and less myelinated structures in all age groups, which resulted in better visualization. There were strong positive correlations between the T1w/T2w ratio values from the majority of white matter ROIs and the subjects’ age (*R* = 0.7–0.9, *p* < 0.001). Within all of the analyzed regions, there were non-linear relationships between age and T1/T2 ratio values that varied by anatomical and functional location. Regions such as the splenium and the genu of the corpus callosum showed the highest R^2^ values, thus indicating less scattering of data and a better fit to the model.

**Conclusion:**

The T1w/T2w mapping technique may enhance our diagnostic ability to assess myelination patterns in the brains of infants and young children.

## Introduction

White matter (WM) is an integral part of the brain’s structure and function. It accounts for more than half of the total brain volume and, like gray matter, is responsible for the motor, sensory, cognitive, and affective functions (Buyanova and Arsalidou, 2021). WM mainly consists of neuronal fibers (axons), which transmit signals between neurons of different CNS regions. As a result of the myelin sheath, nerve impulses are transmitted faster, thus ensuring brain functionality.

The myelin sheath has a very complex biochemical structure and contains cholesterol, lipids (both phospho- and glycolipids), and proteins (Barkovich, 2000). Additionally, it changes composition and compactness during development and maturation, thus leading to signal changes on magnetic resonance imaging (MRI). In general, myelination is associated with T1 and T2 relaxation time shortening (Branson, 2013). With its progression, white matter increases in intensity relative to gray matter on T1-weighted images and decreases in intensity on T2-weighted images (Barkovich, 2000).

White matter myelination is an essential process of CNS maturation. It is believed that this process begins in the fifth fetal month and continues throughout life (Branson, 2013). At birth, only a small portion of the neuronal fibers (such as the dorsal portion of the brainstem, the posterior limb of the internal capsule, and the perirolandic WM) are covered with myelin sheaths. Brain myelination is most active in the first two years of life and subsequently decelerates and progresses at a slow rate until adulthood (van der Knaap and Valk, 2005). Different axons myelinate at different rates and times, which reflects their functional significance. Specific milestones have been established for time points when white matter intensity changes (relative to gray matter intensity) (Barkovich, 2000). In essence, myelination progresses from bottom to top (or caudocranial), posterior to anterior, and central to peripheral (Branson, 2013).

An important part of routine pediatric neuroradiology practice involves the assessment of brain myelination patterns in infants and young children. It is necessary for estimating CNS maturation, as well as for revealing different forms of delays or impairments in myelination (Kühne et al., 2021). In practice, this assessment is usually performed by visually checking T1-weighted images (T1-WI) and T2-weighted images (T2-WI) series (qualitative assessment). However, this approach is highly subjective.

The accurate quantification of myelination processes using neuroimaging techniques is challenging because quantitative MRI techniques are usually time-consuming and have difficult acquisitions (van der Weijden et al., 2021). However, the scanning time is of particular importance in pediatric populations because a patient’s motion can cause a significant issue during the procedure (Saunders et al., 2007). Furthermore, quantitative techniques require high spatial resolution, which depends on acquisition time (Buyanova and Arsalidou, 2021). Thus, to date, there is no consensus about the necessity of the quantification of myelination by increasing the total scan time.

The quantitative assessment of brain myelination has been a significant area of research during the last several decades (Buyanova and Arsalidou, 2021). Several diffusion-tensor imaging metrics, such as fractional anisotropy and mean diffusivity, have been demonstrated to reflect WM changes associated with myelination (Löbel et al., 2009; Morris et al., 2020). Classic diffusion-weighted imaging with the assessment of apparent diffusion coefficient (ADC) values has also been used for this goal (Watanabe et al., 2013; Korostyshevskaya et al., 2019). Low image contrast and low spatial resolution are the serious disadvantages of these techniques, which do not allow for the differentiation of small anatomical structures. In addition, diffusion tensor imaging leads to a significant prolongation of the total scanning time; as a result, this technique cannot be routinely used in the population of young children. There have also been attempts to quantify normal brain myelination by using T1-relaxation rate (R1) (Kühne et al., 2021), magnetization transfer ratio (MTR) (van Buchem et al., 2001), myelin water fraction (MWF) (MacKay and Laule, 2016), and macromolecular proton fraction (MPF) (Yarnykh, 2016; Korostyshevskaya et al., 2019) techniques.

The T1w to T2w signal intensity ratio (or T1w/T2w mapping) is also a neuroimaging method of measuring brain myelination (Glasser and van Essen, 2011). It requires only conventional T1-WI and T2-WI, which are divided on each other voxel-by-voxel after several postprocessing steps (such as spatial coregistration, bias correction, and non-linear intensity normalization); moreover, this algorithm allows us to receive semiquantitative T1w/T2w maps (Ganzetti et al., 2014). The advantages of T1w/T2w mapping include its simplicity and lack of an increase in the total time of MRI acquisition; additionally, this method is plausible for retrospective analyses.

In general, the signal intensity from white matter on T1-weighted images reflects the spatial distribution of myelin-bound cholesterol and glycolipids (Glasser et al., 2014); additionally, it is believed that T1 shortening in developing white matter occurs at a time when the concentration of these molecules is increasing and when they are starting to bind free water molecules (Barkovich, 2000). Conversely, T2w-hypointensity reflects relatively larger myelin content (Glasser et al., 2014). It is believed that the signal intensity from white matter on T2-weighted images is mainly related to the concentration of axonal and extracellular water (Glasser and van Essen, 2011). An increase in T2w-hypointensity in the maturing brain correlates with chemical maturation of the myelin sheath, specifically with the tightening of the spiral around the axon and saturation with some polyunsaturated fatty acids (Barkovich, 2000). Therefore, the T1w to T2w signal intensity ratio should be able to semiquantitatively reflect the myelin content (Ganzetti et al., 2014).

There have been numerous studies dedicated to the application of T1w/T2w mapping in patients with multiple sclerosis (Nakamura et al., 2017), schizophrenia (Iwatani et al., 2015), autism spectrum disorders (Darki et al., 2021), and even spinal cord pathology (Teraguchi et al., 2014). However, despite the high theoretical attractiveness of this technique, there have been only a few published studies regarding its use in infant populations (Soun et al., 2017).

The purpose of this study was to validate the T1w/T2w mapping technique in evaluating brain myelination in infants and young children. We assessed myelination in a relatively large cohort of infants and young children (in which data are sparse to date) with this technique and strived to evaluate its diagnostic utility. Moreover, we aimed to draw attention to the power of quantitative imaging in clinical practice that can be achieved by using only conventional sequences without increasing the total scan time.

## Materials and methods

### Subjects

**FIGURE 1:**
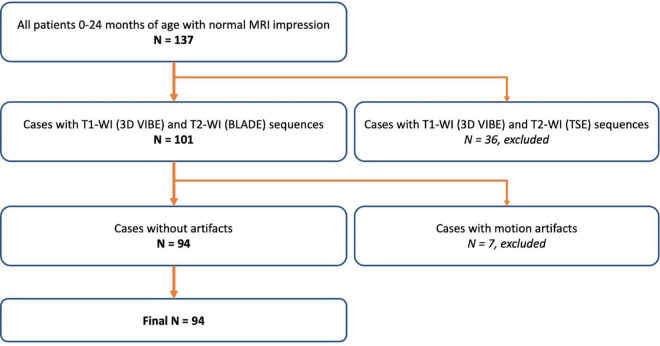
An overview of the patient selection process.

For this retrospective study, the subjects included infants and young children who underwent brain MRI at the Federal Neurosurgical Center, Novosibirsk, from 2013 to 2021. A total of 94 patients (50 boys[and 44 girls; 0–23 months of age; average age: 9.1 ± 6.8 months) participated in the study. All of the patients were born at term. In all of the cases, MRI was performed under general anesthesia with inhalation anesthetic. Brain MRI indications involved uncertain neurosonographic results or clinical suspicion of structural pathology in all of the cases. The main indication for MRI was suspicion of brain developmental anomalies (detailed information is shown in Supplementary File 1). Each case was negative for structural brain pathology and diffusion restriction areas on the brain MRI. Furthermore, cases with motion artifacts were not included in this study (the flow chart for patient selection is shown in Figure 1). The study was approved by the Federal Center of Neurosurgery Novosibirsk institutional ethical committee.

### MRI acquisition

The MR imaging data were acquired by using a 1.5-T system Magnetom Avanto (Siemens, Erlangen, Germany) equipped with a 8-channel receiver head coil; additionally, all of the sessions were performed under general anesthesia. The MRI protocol included T1-WI (3D VIBE), T2-WI (BLADE), FLAIR, DWI, and SWI, and the total acquisition time was approximately 15 minutes. The T1-WI high-resolution sequence (3D VIBE in the axial plane) had the following parameters: TR = 6.26 ms, TE = 2.42 ms, FOV = 256*256 mm, matrix = 256*256, and slice thickness = 1 mm. The T2-WI sequence (BLADE in the axial plane) had the following parameters: TR = 8,500 ms, TE = 138 ms, FOV = 230*230 mm, matrix = 320*320, and slice thickness – 4 mm.

### MRI postprocessing and analysis

This part of the experiment was performed by using the MRTool toolbox (https://www.nitrc.org/projects/mrtool) based on the SPM12 software package (http://www.fil.ion.ucl.ac.uk/spm; Wellcome Department of Imaging Neuroscience, London, UK). The processing steps for T1w/T2w ratio map creation included T1-WI and T2-WI coregistration, bias correction, and intensity normalization by using the linear scaling procedure, as previously described (Ganzetti et al., 2014). The coregistration consisted of two steps: registration of T1-WI and T2-WI independently in MNI space with reslicing to the template’s resolution (1 mm^3^), with subsequent coregistration of T1-WI and T2-WI.

**FIGURE 2:**
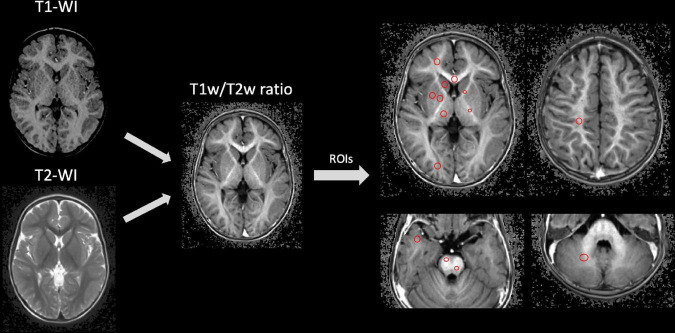
Schematic illustration of MRI data postprocessing.

Further analysis of T1w/T2w ratio maps was performed via ImageJ software (https://imagej.nih.gov/ij/). Round-shaped regions of interest (ROIs) were set at the levels of the dorsal and ventral portions of the pons, cerebellar white matter, splenium and genu of the corpus callosum, anterior and posterior limbs of the internal capsule, central gyri white matter, parietal white matter, white matter of the frontal, temporal, and occipital poles, and head of the caudate, putamen, pallidum, and thalamus. Depending on its anatomical location, the area of the ROI varied from 12 mm^2^ to 20 mm^2^. We averaged the regional values from the right and left hemispheres and analyzed them together. All of the analyses were performed by two certified radiologists (5 years of practice). A schematic representation of the entire MRI postprocessing algorithm is shown in Figure 2.

### Statistical analysis

Statistical analysis was performed by using R software (www.r-project.org). Interrater agreement was calculated with the intraclass correlation coefficient (ICC) by using the ‘psy’ library in R; the specific coefficients for each ROI were calculated, and the minimal value was collected. The presence and type of correlations between regional T1w/Tw values and patient age (in months) were assessed by using the Pearson’s correlation analysis. After identifying a positive correlation between the parameters, we performed a regression analysis. The first step of the regression analysis was performed by using LOESS regression to generally describe the relationships. This analysis demonstrated that quadratic relationships are best suited for most ROIs, and the 2^nd^ order polynomial regression analysis was performed (when appropriate, depending on the distribution of the residuals requirements). Statistical significance was defined as p < 0.05 (with FDR correction).

## Results

**FIGURE 3:**
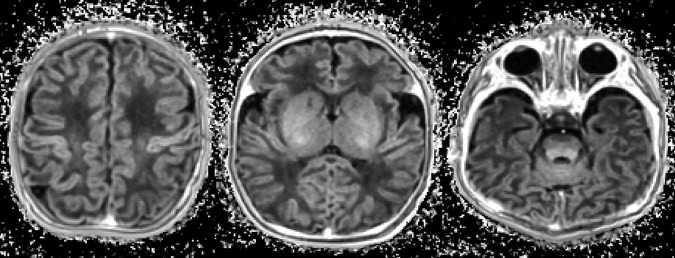
Example of a T1w/T2w map in a neonate (10 days of age).

Neonates showed hyperintensity in areas such as the dorsal pons, the posterior limb of the internal capsule, and the corticospinal tract on T1w/T2w maps compared to other regions. Additionally, regions such as the ventral pons, the corpus callosum, and hemispheric white matter were hypointense in neonates, with an increase in the T1w/T2w ratio values with the maturation of the CNS. These results are consistent with the classic spatiotemporal pattern of brain myelination (van der Knaap and Valk, 2005). Figure 3 demonstrates an example of T1w/T2w maps in a neonate patient.

**FIGURE 4:**
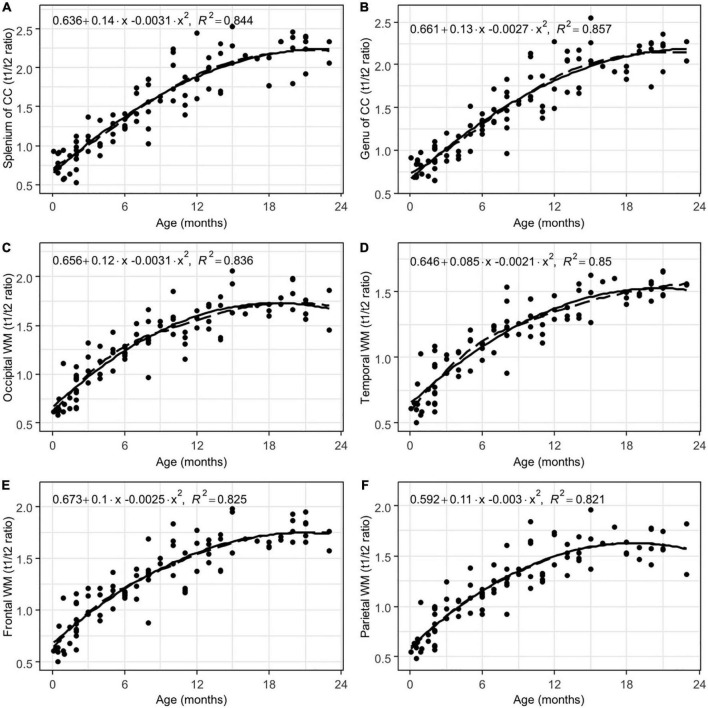
Results of LOESS and 2nd order polynomial regression analyses between subjects’ age (in months) as the predictor variable and the T1w/T2w ratio values as the response variable in the splenium **(A)** and the genu **(B)** of the corpus callosum, as well as the occipital **(C)**, temporal **(D)**, and frontal **(E)** poles and parietal **(F)** white matter. The dotted line represents the LOESS regression curve, and the solid line represents the 2nd-order polynomial regression curve (adjusted *p* < 0.001).

**FIGURE 5:**
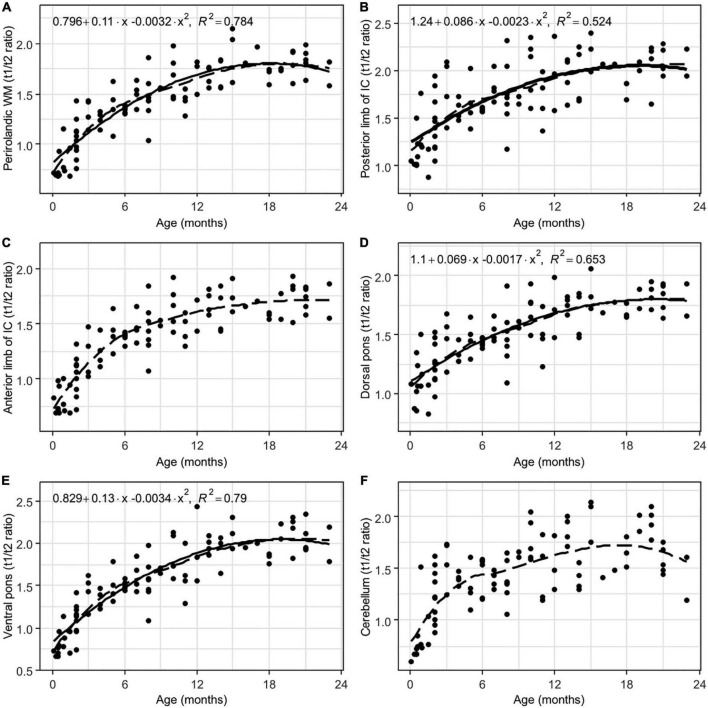
Results of LOESS and 2nd order polynomial regression analyses between subjects’ age (in months) as the predictor variable and the T1w/T2w ratio values as the response variable in perirolandic white matter **(A)**, the posterior **(B)** and the anterior **(C)** limbs of the internal capsule, the dorsal **(D)** and the ventral **(E)** parts of the pons, and cerebellum white matter **(F)**. The dotted line represents the LOESS regression curve, and the solid line represents the 2nd-order polynomial regression curve (adjusted *p* < 0.001).

**FIGURE 6:**
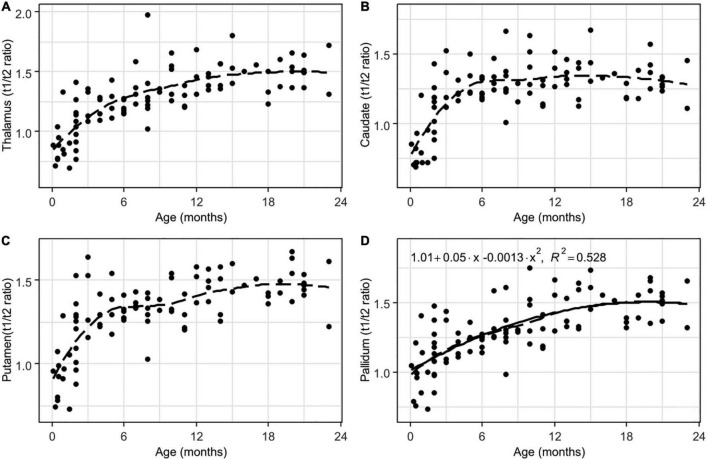
Results of LOESS and 2nd order polynomial regression analyses between subjects’ age (in months) as the predictor variable and the T1w/T2w ratio values as the response variable in thalamus **(A)**, the head of the caudate nucleus **(B)**, putamen **(C)**, and pallidum **(D)**. The dotted line represents the LOESS regression curve, and the solid line represents the 2nd-order polynomial regression curve (adjusted *p* < 0.001).

Interrater agreement was excellent (ICC = 0.91). There were significant positive correlations between the subjects’ age and the T1w/T2w ratio regional values from all of the analyzed white matter ROIs (adjusted *p* < 0.001, FDR corrected, Table 1). Moreover, highly non-linear relationships between the parameters were observed in all of the ROIs according to the LOESS regression, with almost linear growth being observed in the first 6–9 months of life, after which decreased growth was observed. These relationships were well described by a 2nd-order polynomial regression for most of the analyzed regions (adjusted *p* < 0.001, FDR corrected, Table 1 and Figures 4–6).

**TABLE 1:** Results of Pearson’s correlation analysis and 2nd order polynomial regression analysis between children’s age and the T1w/T2w ratio values.

Early myelinated regions						
	**Perirolandic WM**	**Dorsal pons**	**Ventral pons**	**Cerebellum**	**Posterior limb of IC**	**Anterior limb of IC**
Pearson’s test results (FDR corrected)	*R*=0.82	*R*=0.77	*R*=0.84	*R*=0.62	*R*=0.68	*R*=0.65
2nd order polynomial regression results (FDR corrected)	0.80+ 0.11.x –0.0032.x^2^ *R*^2^ = 0.784	1.1 + 0.069.x –0.0017.x_2_ *R*2 = 0.653	0.829+ 0.13.x –0.0034.x^2^ R^2^ = 0.79	NA	1.24+ 0.086.x –0.0023.x^2^ R^2^ = 0.524	NA
**Late myelinated regions**						
	**Splenium of CC**	**Genu of CC**	**Frontal pole WM**	**Parietal pole WM**	**Temporal pole WM**	**Occipital pole WM**
Pearson’s test results (FDR corrected)	R=0.89	R=0.9	R=0.87	R=0.85	R=0.88	R=0.86
2nd order polynomial regression results (FDR corrected)	0.636+ 0.14.x –0.0031.x^2^ R^2^ = 0.844	0.661+ 0.13.x –0.0027.x^2^ R^2^ = 0.857	0.673+ 0.1.x –0.0025.x^2^ R^2^ = 0.825	0.592+ 0.11.x –0.003.x^2^ R^2^ = 0.821	0.646+ 0.085.x –0.0021.x^2^ R^2^ = 0.85	0.656+ 0.12.x –0.0031.x^2^ R^2^ = 0.836
**Thalamus and basal ganglia**						
	**Thalamus**	**Head of caudate**	**Putamen**	**Pallidum**		
Pearson’s test results (FDR corrected)	R=0.71	R=0.51	R=0.61	R=0.69		
2nd order polynomial regression results (FDR corrected)	NA	NA	NA	1.01+ 0.05.x –0.0013.x^2^ R^2^ = 0.528		

Adjusted *p* < 0.001 for all measurements. CC, corpus callosum; IC, internal capsule; WM, white matter. NA, not applicable (data did not show a normal distribution of residuals).

Based on the performed analysis, regions such as the splenium and the genu of the corpus callosum showed the highest coefficient of determination (R^2^) values, thus indicating less scattering of data and a better fit to the model. The T1w/T2w ratio values within regions such as the anterior limb of the internal capsule, cerebellum, thalamus, caudate, and putamen did not exhibit a normal distribution of residuals, which indicated that a different type of non-linear regression analysis was needed. Furthermore, all of the T1w/T2w ratio regional values for each patient are shown in Supplementary File 2.

## Discussion

In conjunction with our hypothesis, we observed statistically significant positive correlations between regional T1w/T2w ratio values and patient age. However, the relationships between age and T1w/T2w ratio values were non-linear and well described via a parabolic function. During the first six to nine months of life, T1w/T2w ratio values showed the greatest changes, thus suggesting the most active period of myelination.

The greatest changes in the T1w/T2w ratio values with age were found in regions that begin to myelinate after birth, such as the splenium and the genu of the corpus callosum, as well as the hemispheric WM. In the first two years of life, the T1w/T2w ratio values can increase by approximately five times in these regions. This suggests a high rate of myelination processes. Moreover, the T1w/T2w ratio values appear to be relatively stable throughout the different age groups in late-myelinating structures, especially in the splenium and the genu of the corpus callosum. Moreover, there was less scattering of data and a better fit to our regression model; additionally, the regression curves were observed to be the steepest. Thus, the T1w/T2w ratio values from the splenium and the genu of the corpus callosum may be used as a reference when compared to pathological states. This effect could be a subject for further research.

Temporal pole and frontal pole WM had the lowest absolute values of T1w/T2w signal intensity in all of the age groups. These results confirm that these regions are late-myelinating. It is well known that there are terminal zones of myelination in these areas (Branson, 2013).

There were less pronounced correlations between age and the T1w/T2w ratio values in the early myelinated regions, such as the pons and corticospinal tract projections. At birth, these regions are partially myelinated. Consequently, it is not surprising that their myelination processes are slower.

Interestingly, the relationships between age and T1w/T2w ratio values in the thalamus and basal ganglia were also non-linear (but not well described by using a parabolic function). This indicates that even though gray matter undergoes myelination, it follows a slightly different dynamic.

Our results fully confirm the assumption that has been outlined by the authors of the T1w/T2w mapping technique (Ganzetti et al., 2014). The spatial-temporal changes in signal intensity on T1w/T2w ratio images occur in posterior-to-anterior and caudal-to-rostral directions (Figures 2–4) and match the well-known pattern of normal brain myelination.

For several reasons, we decided not to assess the T1w/T2w ratio values in the cerebral cortex in this study. First, it is extremely difficult to manually align placed ROIs in cortical regions across all of the subjects; unfortunately, there are a limited number of tools for automatic brain segmentation in infants. Additionally, there was a relatively low resolution of calculated maps for this goal. Nonetheless, it would be interesting to examine whether spatiotemporal patterns of cortical maturation correlate with WM myelination. Therefore, this possibility may be the subject of further investigations.

There are several advantages of T1w/T2w mapping compared to other neuroimaging techniques (such as DTI, MTR, and MWF). First, there is no need to increase the total scanning time because the techniques rely on postprocessing T1-WI and T2-WI, which is an aspect of the routine brain MRI acquisition protocols (Glasser and van Essen, 2011). Second, the maps’ visual interpretations are intuitive, and the postprocessing procedure is relatively straightforward. Finally, the resulting maps have a spatial resolution of equally high quality as native T1-WI (Ganzetti et al., 2014). In addition, the majority of other methods are difficult to use in routine clinical practice due to technical issues (such as sophisticated image acquisitions and/or long scanning times), especially in the pediatric population. Conversely, the T1w/T2w mapping technique could be highly useful in routine pediatric neuroradiology practice.

An additional promising method for assessing childhood brain myelination is T1 mapping (Kühne et al., 2021). This method has a common theoretical background with T1w/T2w mapping. Moreover, it is based on the signal calculation from white matter on postprocessed T1-maps, which reflects the spatial distribution of myelin-bound cholesterol. The disadvantage of this technique involves the low availability of retrospective data analysis, due to the fact that T1-WI sequences that are applicable for T1 mapping are rarely used in routine MRI acquisition protocols.

One more promising method of myelination assessment is macromolecular proton fraction mapping (Yarnykh, 2016). This technique has several clear advantages compared with T1w/T2w mapping, such as the ability to quantify brain myelin content in absolute values and a higher standardization of values. Unfortunately, limited availability (due to the fact that it is a non-commercial product) is the main limitation of this method.

To the best of our knowledge, this was the first attempt to assess normal brain myelination in infants and young children by using the T1w/T2w mapping technique. In one previously published study, the usefulness of this method was demonstrated in neonates (Soun et al., 2017). The authors showed that T1w/T2w ratio images have superior visual contrast between different brain regions compared to T1-WI, T2-WI, and ADC maps; our results also confirm this statement.

This study also had several limitations. First, although all of the patients in our study did not have structural brain pathology, our group of patients was not representative of a normal population. Second, it was impossible to compare the T1w/T2w mapping technique with other quantitative MRI techniques in our study.

Further studies in this area with more sample sizes and additional usage of the methods of automatic image segmentation are needed to prove our results. Additionally, it will be interesting to compare different methods of myelination assessment, as well as compare the patients with some myelination impairments to controls.

## Conclusion

The T1w/T2w ratio is a straightforward method to measure white matter integrity, which is strongly correlated with the subjects’ age during the first two years of life. According to our findings, the T1w/T2w mapping technique may be useful in assessing myelination in infants and young children.

## Data availability statement

The raw data supporting the conclusions of this article will be made available by the authors, without undue reservation.

## Ethics statement

The studies involving human participants were reviewed and approved by Federal Neurosurgical Center Novosibirsk Ethics Committee. Written informed consent to participate in this study was provided by the participants’ legal guardian/next of kin.

## Author contributions

EF, EA, and JR made general study design and wrote the manuscript text. EA performed the statistical analysis. EF, BZ, and AS performed the MRI scans analysis and postprocessing. All authors contributed to the article and approved the submitted version.
